# Pericardial fat volume and coronary risk factors as predictors of non-calcified coronary plaque presence among patients with coronary calcium score = 0

**DOI:** 10.1016/j.ihj.2023.12.006

**Published:** 2023-12-19

**Authors:** Abdulameer A. Al-Mosawi, Hussein Nafakhi, Yusra Sahib Alabayechi

**Affiliations:** a Radiology Department, Medicine College, University of Kufa, Najaf, Iraq; b Internal Medicine Department, Medicine College, University of Kufa, P.O. 21, Kufa, Najaf, Iraq; c Al-Sader Teaching Medical City, Najaf Health Directorate, Najaf, Iraq

**Keywords:** Coronary, Non-calcified plaque, Pericardial fat

## Abstract

**Introduction:**

There is scarce data linking pericardial fat volume (PFV) and classical coronary risk factors with non-calcified plaque presence among patients with CAC = 0 in the literature.

**Material and method:**

A total of 811 patients with chest pain suggestive of angina underwent CT coronary angiography for the assessment of coronary artery disease were collected. Of these, 417 with CAC = 0 were included in the analysis.

**Result:**

Patients with non-calcified plaque were older (54 ± 9 versus 50 ± 10, *P* = 0.01) and had a higher prevalence of diabetes mellitus (31% versus 17%, *P* = 0.02), high BMI (29.9 versus 28.3, *P* = 0.04), and increased PFV (123 cm^3^ versus 99 cm^3^, *P* < 0.01) compared to patients without plaque. In multivariate regression analysis, high BMI[OR(CI) = 1.1(1–1.3), *P* = 0.02] was an independent predictor of non-calcified coronary plaque presence among patients with CAC = 0 after adjustment to variables with *P* < 0.05 in the univariate analysis.

**Conclusion:**

In patients with a CAC score of 0, advanced age, diabetes mellitus, increased PFV, and high BMI were all associated with the presence of non-calcified plaque. After multivariate adjustment, increased BMI remained a significant independent predictor for non-calcified plaque presence.

## 1 Introduction

Coronary artery calcium score (CAC) = 0 in patients with low-intermediate risk for coronary artery disease (CAD) confers a low risk for morbidity and mortality after 10 years of follow-up, as reported in large cohort studies.^1^, ^2^, ^3^ However, CAC = 0 does not consider the presence of non-calcified plaques, which cannot be detected by conventional angiography or routine CAC scan, that may have prognostic implications when identified by CT coronary angiography.^3^, ^4^, ^5^, ^6^

The early stage of coronary atherosclerosis involves the formation of non-calcified coronary plaque, which is linked with increased shear stress and positive remodeling of the coronary vessel.^7^^,^^8^ This type of coronary plaque is more liable to rupture and thrombus formation, likely causing acute coronary syndrome or even sudden cardiac death in comparison to calcified plaque, which represents a more stable lesion and a late phase of coronary atherosclerosis.^7^, ^8^, ^9^ Hence, identifying the potential predictors of non-calcified plaque is crucial for risk stratification and risk factor control in patients with suspected CAD, particularly when CAC = 0.

Recently, several clinical reports have suggested that increased pericardial fat deposition can predict the risk of CAD regardless of CAC score.^10^^,^^11^ However, there is scarce data linking pericardial fat volume (PFV) and classical coronary risk factors with non-calcified plaque presence in patients with CAC = 0 in the literature. The present study aimed to assess the association of PFV and classical coronary risk factors with non-calcified plaque presence in patients with suspected CAD and CAC = 0.

## 2 Methods

This retrospective study was conducted between January 2013 and April 2022. A total of 811 patients with stable chest pain suggestive of angina who had an intermediate probability of CAD and were referred for multi-detector computed tomography (MDCT) examination to exclude the presence of occlusive CAD were collected. Of these, 417 with CAC = 0 were included in the analysis. The clinical and demographic characteristics were obtained at the time of MDCT examination as in our prior study.^12^ Verbal informed consent was obtained from each patient before inclusion in the study.

### 2.1 MDCT examination

A 64-slice scanner equipped with electrocardiogram (ECG) gating was employed to perform coronary angiography (Aquilon 64, v. 4.51 ER 010; Toshiba Medical Systems). Also, a computed tomography scan without contrast dye with 3 mm slice thickness was used to calculate the CAC using the Agatston method. The amount of fat tissue located inside the pericardial sac, excluding fat outside the parietal pericardium, was measured three-dimensionally using contrast-enhanced phase imaging. Non-calcified (soft) plaque was defined as a discernible structure ≥1 mm in thickness free of calcium within or adjacent to the coronary artery wall, with a CT number below the contrast-enhanced coronary lumen but above the surrounding connective tissue.^3^^,^^12^

Two radiologists, both experienced in interpreting coronary MDCT angiography with more than 5 years' experience, carried out all the data analysis independently.

### 2.2 Statistical analysis

Categorical variables were represented numerically as percentages and continuous variables as mean ± standard deviation (SD). The unpaired *t*-test was used to compare continuous variables, while the chi-square test was employed for categorical variables. Univariate and multivariate regression analyses were performed to identify the predictors of non-calcified plaque presence among patients with CAC = 0. Those variables that had *P* < 0.05 in univariate analysis were then entered into the multivariate binary regression analysis. A two-tailed *P*-value of <0.05 indicates statistical significance. All of the statistical analyses were conducted using SPSS ver. 17.0 (SPSS Inc., Chicago, Illinois).

## 3 Results

A total of 417 patients with CAC = 0 were included in the analysis. Patients were then divided into 2 groups: those with non-calcified plaque group[*n* = 48(11.5%)], and those without plaque group[*n* = 369(88.5%)]. Patients with non-calcified plaque were older (54 ± 9 versus 50 ± 10, *P* = 0.01) and had a higher prevalence of diabetes mellitus (31% versus 17%, *P* = 0.02), high BMI (29.9 versus 28.3, *P* = 0.04), and increased PFV (123 cm^3^ versus 99 cm^3^, *P* < 0.01) compared to patients without plaque. The patients^,^ clinical characteristics are shown in Table 1.

**Table 1:** Patients’ characteristics.

	Non-calcified plaque group *n* = 48 mean ± SD or n (%)	Without plaque group *n* = 369 mean ± SD or n (%)	*P* value
Age (years)	54 ± 9	50 ± 10	0.01
Male	21(44%)	160(43%)	0.55
Hypertension	25(52%)	179(48%)	0.46
Diabetes mellitus	15(31%)	63(17%)	0.02
Smoking	12(25%)	58(16%)	0.09
Family history	15(31%)	76(20%)	0.08
BMI	29.9 ± 5	28.3 ± 5	0.04
PFV (cm^3^)	123 ± 48	99 ± 55	<0.01
Normal to non-significant coronary stenosis <50%	44(92%)	–	–
Significant coronary stenosis ≥50%	4(8%)	–	–

BMI = body mass index, PFV = pericardial fat volume, SD = standard deviation.

### 3.1 Multivariate analysis

In multivariate binary regression analysis, high BMI[OR(CI) = 1.1(1–1.3), *P* = 0.02] was an independent predictor of non-calcified coronary plaque presence among patients with suspected CAD and CAC = 0 after adjustment to variables with *P* < 0.05 in univariate analysis, as in Table 2.

**Table 2:** Multivariate regression analysis*.

Non-calcified plaque			
	OR	CI	*P* value
Age	1	0.9–1.1	0.07
Diabetes mellitus	1.6	0.7–3	0.20
BMI	1.1	1–1.3	0.02
PFV	1	0.9–1.1	0.24

BMI = body mass index, CI = confidence interval, OR = odds ratio, PFV = pericardial fat volume.

*Variables with *P* < 0.05 in univariate analysis were entered into the multivariate binary regression analysis.

## 4 Discussion

In the literature, the relationship between pericardial fat and non-calcified plaque among patients with CAC = 0 has yet to be thoroughly investigated, and the findings have been inconsistent.^13^

Pericardial fat adipocytes secret several adipokines and cytokines and stimulate macrophage cells to infiltrate the coronary vessel wall. Such active inflammatory changes are commonly observed in the pericardial fat of patients with acute coronary events. On the other hand, patients with stable CAD, like our sample population, may have mild inflammation with less macrophage infiltration as well as a thick fibrous cap on the surface of the coronary plaque. Thus, plaque burden in patients with acute coronary syndrome or sudden cardiac death is often higher than that in stable CAD, and the association of PFV with plaque burden may vary depending on the stage of CAD.^11^^,^^14^

Supporting this notion, increased PFV was positively correlated with non-calcified plaque burden in patients with acute coronary syndrome but not in stable CAD.^11^ The authors have suggested that the effect of pericardial fat deposition on plaque composition may be more than a single mechanism, and PFV may have differential effects on non-calcified plaque depending on the phase of coronary atherosclerosis.^11^

Also, increased PFV was associated with non-calcified plaque presence among obese patients with suspected CAD but not in normal-weight patients in our previous work, suggesting a differential relationship of increased PFV with coronary atherosclerotic plaques among different BMI grades.^12^

Taken together, PFV may have differential effects on non-calcified plaque depending on the phase of coronary atherosclerosis, the clinical presentation of the sample population (symptomatic Vs. asymptomatic, acute Vs. stable CAD), the biological and inflammatory status of pericardial fat, potential confounders or comorbidities, and CAC status.

Regarding BMI and non-calcified plaque, Gullaksen S et al found a significant association between BMI with non-calcified plaque even after adjusting for age, sex, pericardial fat, and diabetes status. The authors proposed that BMI could be related to coronary atherosclerosis through other pathways which could be caused by clustering of high-risk factors such as hypertension and diabetes mellitus.^15^

The present study has several limitations. First, it was a retrospective and single-center study. Second, plaque location and high risk characteristics were not assessed. Third, there was a lack of data on lipid profile or specific lipid-lowering medications for the majority of participants. Fourth, we did not assess inflammatory markers or adipokines in our study, which could be relevant for understanding coronary atherosclerotic plaque burden and assessing the inflammatory and biological effects of pericardial fat. Fifth, the total number of non-calcified plaque was relatively small may resulting in reduced power to detect the significant statistical differences in multivariate regression analysis.

## 5 Conclusion

The presence of non-calcified plaque in patients with a CAC score of 0 was associated with advanced age, diabetes mellitus, increased PFV, and high BMI. After multivariate adjustment, BMI remained a significant independent predictor for the presence of non-calcified plaque.

## Funding

There were no external funding sources for this study.

## Declaration of competing interest

The authors declare that they have no known competing financial interests or personal relationships that could have appeared to influence the work reported in this paper.

## References

[1] Bernardini F., Gelfusa M., Celeski M. (2023). Beyond the calcium score: what additional information from a CT scan can assist in cardiovascular risk assessment?. Appl Sci.

[2] Senoner T., Plank F., Beyer C. (2020). Does coronary calcium score zero reliably rule out coronary artery disease in low-to-intermediate risk patients? A coronary CTA study. J Cardiovasc Comput Tomogr.

[3] Iwasaki K., Matsumoto T., Aono H., Furukawa H., Samukawa M. (2010). Prevalence of non-calcified coronary plaque on 64-slice computed tomography in asymptomatic patients with zero and low coronary artery calcium. Can J Cardiol.

[4] Stojan G., Li J., Budoff M., Arbab-Zadeh A., Petri M.A. (2020). High-risk coronary plaque in SLE: low-attenuation non-calcified coronary plaque and positive remodelling index. Lupus Sci Med.

[5] Hwang I.C., Park H.E., Choi S.Y. (2017). Epicardial adipose tissue contributes to the development of non-calcified coronary plaque: a 5-year computed tomography follow-up study. J Atherosclerosis Thromb.

[6] Al-Muhaidb S.M., Aljebreen A.M.M., AlZamel Z.A., Fathala A. (2021). Prevalence of noncalcified plaques and coronary artery stenosis in patients with coronary calcium scores of zero. Coron Artery Dis.

[7] Bauer R.W., Thilo C., Chiaramida S.A., Vogl T.J., Costello P., Schoepf U.J. (2009). Noncalcified atherosclerotic plaque burden at coronary CT angiography: a better predictor of ischemia at stress myocardial perfusion imaging than calcium score and stenosis severity. AJR Am J Roentgenol.

[8] Sahara M., Kirigaya H., Oikawa Y. (2004). Soft plaque detected on intravascular ultrasound is the strongest predictor of in-stent restenosis: an intravascular ultrasound study. Eur Heart J.

[9] Senoo A., Kitagawa T., Torimaki S. (2018). Association between histological features of epicardial adipose tissue and coronary plaque characteristics on computed tomography angiography. Heart Ves.

[10] Yamaura H., Otsuka K., Ishikawa H., Shirasawa K., Fukuda D., Kasayuki N. (2022). Determinants of non-calcified low-attenuation coronary plaque burden in patients without known coronary artery disease: a coronary CT angiography study. Front Cardiovasc Med.

[11] Shan D., Dou G., Yang J. (2021). Epicardial adipose tissue volume is associated with high risk plaque profiles in suspect CAD patients. Oxid Med Cell Longev.

[12] Hassan M.B., Nafakhi H., Al-Mosawi A.A. (2020). Pericardial fat volume and coronary atherosclerotic markers among body mass index groups. Clin Cardiol.

[13] Gullaksen S., Funck K.L., Laugesen E., Hansen T.K., Dey D., Poulsen P.L. (2019). Volumes of coronary plaque disease in relation to body mass index, waist circumference, truncal fat mass and epicardial adipose tissue in patients with type 2 diabetes mellitus and controls. Diabetes Vasc Dis Res.

[14] Iacobellis G., Barbaro G. (2008). The double role of epicardial adipose tissue as pro- and anti-inflammatory organ. Horm Metab Res.

[15] Gullaksen S., Funck K.L., Laugesen E., Hansen T.K., Dey D., Poulsen P.L. (2019). Volumes of coronary plaque disease in relation to body mass index, waist circumference, truncal fat mass and epicardial adipose tissue in patients with type 2 diabetes mellitus and controls. Diabetes Vasc Dis Res.

